# Prevalence of Hyperuricemia and Gout in Mainland China from 2000 to 2014: A Systematic Review and Meta-Analysis

**DOI:** 10.1155/2015/762820

**Published:** 2015-11-10

**Authors:** Rui Liu, Cheng Han, Di Wu, Xinghai Xia, Jianqiu Gu, Haixia Guan, Zhongyan Shan, Weiping Teng

**Affiliations:** Department of Endocrinology and Metabolism, The Endocrine Institute and The Liaoning Provincial Key Laboratory of Endocrine Diseases, The First Affiliated Hospital of China Medical University, Shenyang, Liaoning 110001, China

## Abstract

We systematically identified the prevalence of hyperuricemia and gout in mainland China and provided informative data that can be used to create appropriate local public health policies. Relevant articles from 2000 to 2014 were identified by searching 5 electronic databases: PubMed, Google Scholar, Chinese Wanfang, CNKI, and Chongqing VIP. All of the calculations were performed using the Stata 11.0 and SPSS 20.0 software. The eligible articles (*n* = 36; 3 in English and 33 in Chinese) included 44 studies (38 regarding hyperuricemia and 6 regarding gout). The pooled prevalence of hyperuricemia and gout was 13.3% (95% CI: 11.9%, 14.6%) and 1.1% (95% CI: 0.7%, 1.5%), respectively. Although publication bias was observed, the results did not change after a trim and fill test, indicating that that impact of this bias was likely insignificant. The prevalence of hyperuricemia and gout was high in mainland China. The subgroup analysis suggested that the geographical region, whether the residents dwell in urban or rural and coastal or inland areas, the economic level, and sex may be associated with prevalence.

## 1. Introduction

Serum uric acid is the final enzymatic product of purine metabolism [1, 2]. Abnormalities in serum uric acid metabolism may cause hyperuricemia and gout. Hyperuricemia is the result of interactions among multiple factors, including sex, age, genetics, lifestyle, and environment [3]. Several studies have suggested that hyperuricemia is associated with many diseases, including diabetes mellitus [4], hypertension [5, 6], stroke [2, 7], dyslipidemia [8], chronic kidney disease [9], cardiovascular events, and heart failure [10–12]. Hyperuricemia is considered to be a precursor of gout as the deposition of urate crystals in the joints results in an acute inflammatory response. Deposition in the soft tissue can lead to tophi [13–15]. Gout is also a serious health issue and is an independent risk factor for heart failure and metabolic syndrome [16, 17]. In recent years, an increasing trend in the prevalence of hyperuricemia and gout has been observed in epidemiological studies [13, 18–21], and both diseases have become public health problems that need to be solved quickly.

Due to rapid economic development, the lifestyle of the Chinese has changed greatly, a huge transition from a dietary pattern traditionally based on carbohydrates and vegetables to a pattern that relies on meat, dairy products, and other purine-rich foods that are closely related to hyperuricemia and gout [22, 23].

As a large developing country, China has marked regional differences and varied populations. To date, most investigations have been limited to certain areas or have focused on specific occupations. Therefore, a comprehensive study on the epidemiology of hyperuricemia and gout in the entire mainland China is needed. As most of the published data are in Chinese, we present our study in the widely read English medium. Obtaining an accurate prevalence of hyperuricemia and gout is important to help us formulate appropriate local public health policies. In addition, such a study will benefit the people through health education by increasing awareness of hyperuricemia and gout and also the importance of improving lifestyle and maintaining a healthy diet.

Due to varied geographic locations that include diverse populations and different socioeconomic conditions, a unified epidemiological investigation about the prevalence of hyperuricemia and gout remains difficult. We conducted a meta-analysis regarding the prevalence of both diseases in mainland China between January 2000 and December 2014 to determine the epidemiology and to review the results from previous studies.

## 2. Methods

### 2.1. Search Strategy

We manually searched all of the literatures regarding population-based research on the prevalence of hyperuricemia and gout from 2000 to 2014 using the PubMed, Google Scholar, CNKI (Chinese National Knowledge Infrastructure), Chinese Wangfang, and Chongqing VIP electronic databases. The keywords for search were “uric acid,” “HUA,” “HU,” “hyperuricemia,” “gout,” “prevalence(s),” “incidence(s),” and “epidemiology.” To find additional studies, the reference lists of the identified studies were also examined.

### 2.2. Inclusion and Exclusion Criteria

Papers were included if they met all of the following criteria: (1) all study participants living in mainland China; (2) study data being general population-based rather than hospital-based; (3) prevalence rate being also analyzed by according to sex; (4) accurate diagnostic criteria and clear study date; and (5) the most detailed study of duplicate studies on the same population.

Studies were excluded if they (1) were not original research, such as a review or case report, (2) included participants with concomitant diseases or had medication history known to affect uric acid metabolism, or (3) focused on specific population groups, such as teenagers, elderly people, or single gender, or a certain occupation.

### 2.3. Definition of Hyperuricemia and Gout

The diagnostic criteria for hyperuricemia varied among the studies; we have listed each criterion in Table 1. The diagnostic criteria for gout were listed in Table 2 [24, 25].

### 2.4. Data Extraction

**Figure 1:**
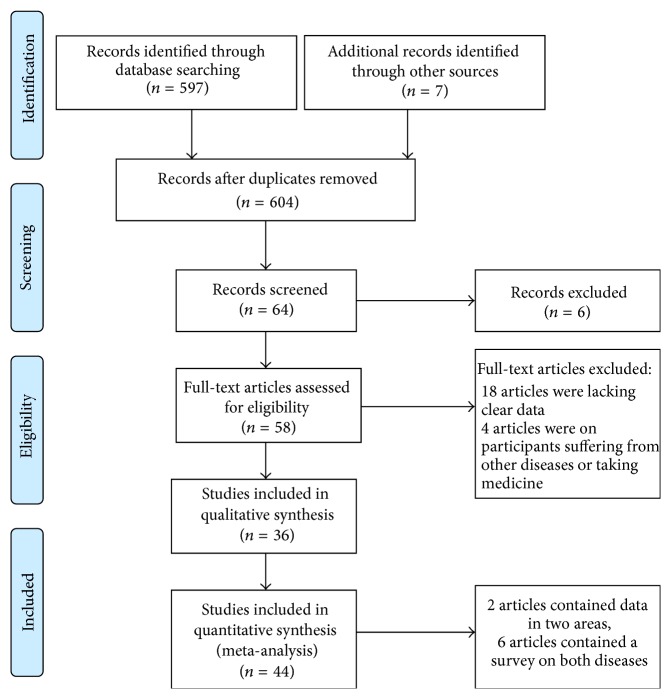
Flow diagram for the literature-search process.

Two reviewers searched the literature independently. Any disagreement on data extraction between the two reviewers was mediated by discussion [26].  Figure 1 shows the literature-search process. We recorded the characteristics of all the included papers in Table 1, including the title, author's name, publication date, study year, study population, geographic area, rural/urban, inland/coastal, sample size, case, prevalence, and diagnostic criterion.

### 2.5. Statistical Analysis

Pooled prevalence and 95% confidence intervals (CIs) were calculated to estimate the prevalence of hyperuricemia and gout in mainland China. We adopted the Chi-squared-based *Q* test and the *I* ^2^ test to evaluate the heterogeneity of the studies; 25%, 50%, and 75% were considered low, moderate, and high levels, respectively [27, 28]. If the level of heterogeneity was moderate or high, we used a random-effects meta-analysis model instead of a fixed-effects model. To perform a secondary analysis and to address heterogeneity, a subgroup analysis was required. Egger's test was used to estimate publication bias. A *P* value less than 0.05 was considered statistically significant. Meta-analysis was calculated using Stata Version 11.0 (Stata Corp LP, College Station, TX, USA). Significant differences in prevalence among the groups were examined through the Chi-square test using SPSS Version 20.0 (SPSS Software, Chicago, IL, USA). All figures were generated using Stata 11.0 (Stata Corp LP, College Station, TX, USA) or Microsoft PowerPoint (Microsoft, Redmond, USA).

## 3. Results

### 3.1. Characteristics of Included Studies

A total of 604 articles were identified (Figure 1). After screening for population base, study type, relevancy, and duplicates, 36 literary papers (3 in English and 33 in Chinese) containing 44 studies (38 regarding hyperuricemia and 6 regarding gout) met our inclusion criteria. A detailed description of these studies is provided in Table 1.

### 3.2. Pooled Prevalence of Hyperuricemia and Gout

**Figure 2:**
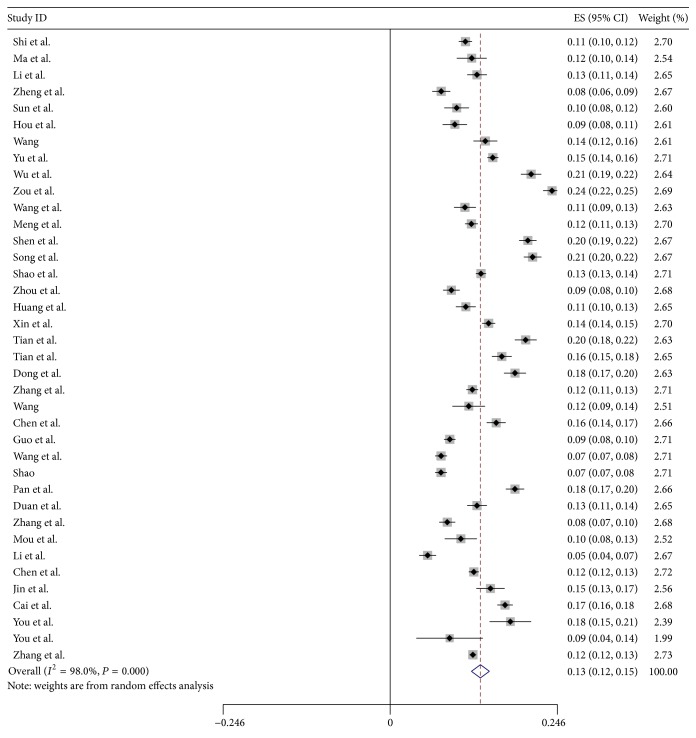
Forest plot of the pooled prevalence of hyperuricemia in mainland China.

**Figure 3:**
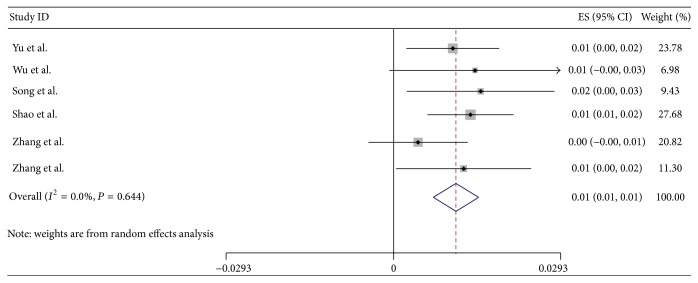
Forest plot of the pooled prevalence of gout in mainland China.

As shown in Figure 2, the pooled prevalence of hyperuricemia was 13.3% (95% CI: 11.9%, 14.6%), with the prevalence ranging from 5.5% to 23.6%. As shown in Figure 3, the pooled prevalence of gout was 1.1% (95% CI: 0.7%, 1.5%), with a range of 0.4–1.5%.

**Figure 4:**
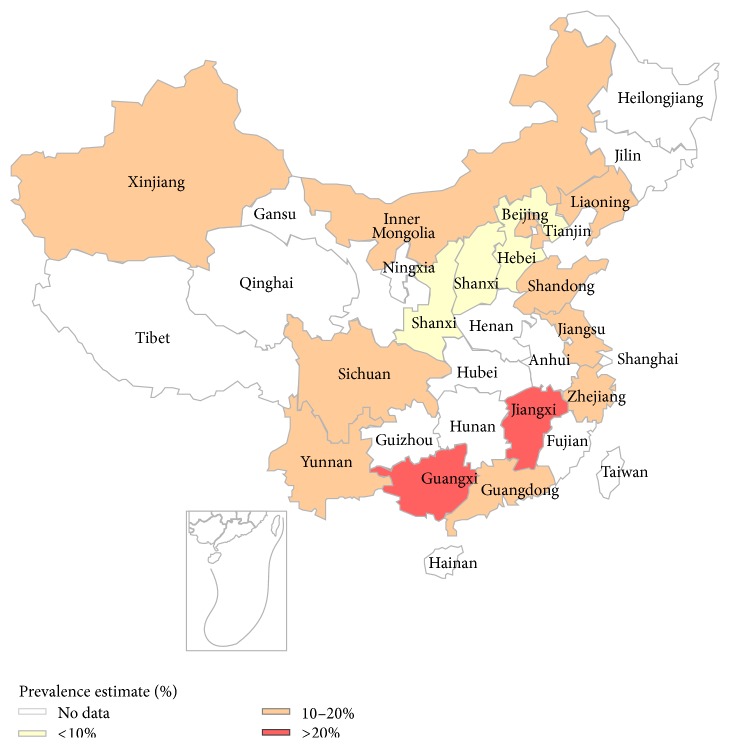
Regional distribution of pooled prevalence of hyperuricemia in mainland China.

**Figure 5:**
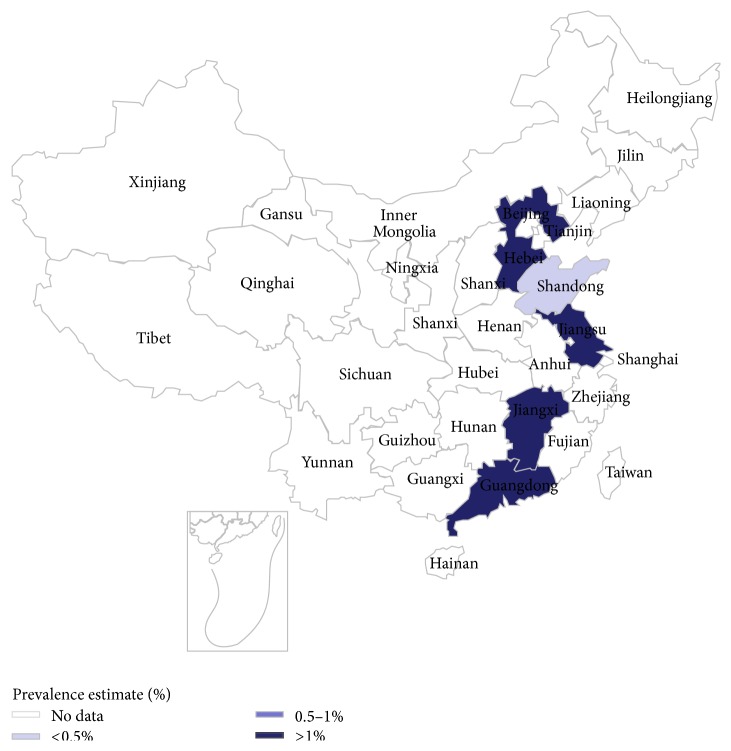
Regional distribution of pooled prevalence of gout in mainland China.

Figures 4 and 5 showed the individual prevalence of hyperuricemia and gout, respectively, in different provinces, municipalities, and autonomous regions.

### 3.3. Subgroup Analysis

The prevalence of hyperuricemia in mainland China was analyzed in subgroups, which were separated based on the following categories: rural or urban, coast or inland, location (north, south, northwest, northeast, and southwest China), economic level, and sex. As shown in Table 3, location in an urban area (*χ* ^2^ = 25.53, *P* < 0.001), inland area (*χ* ^2^ = 117.95, *P* < 0.001), or south China (*χ* ^2^ = 507.39, *P* < 0.001) and a high economic level (*χ* ^2^ = 8.40, *P* = 0.004) might indicate a high prevalence of hyperuricemia. Notably, sex may also be closely associated with hyperuricemia prevalence, as the prevalence among men and women was 19.4% (95% CI: 17.6%, 21.1%) and 7.9% (95% CI: 6.6%, 9.3%), respectively.

For gout, the prevalence among the subgroups was very different (Table 4). Urban residents had a much higher prevalence of gout (1.2%, 95% CI: 0.7%, 1.8%) compared with rural residents (0.9%, 95% CI: 0.2%, 1.6%; *χ* ^2^ = 19.96, *P* < 0.001). Inland area residents had a higher prevalence of gout (1.4%, 95% CI: 0.8%, 1.9%) than coastal area residents (0.8%, 95% CI: 0.2%, 1.4%; *χ* ^2^ = 23.88, *P* < 0.001). An increasing prevalence of gout was seen over the years; 0.9% (95% CI: 0.0%, 1.8%) of subjects investigated from 2000 to 2005 were diagnosed with gout, and this number increased to 1.4% (95% CI: 0.5%, 2.2%) after 2010 (*χ* ^2^ = 7.47, *P* = 0.024). Regarding sex, the prevalence rate was 1.5% (95% CI: 0.8%, 2.1%) in men and 0.9% (95% CI: 0.0%, 1.0%) in women.

### 3.4. Analysis of Heterogeneity and Publication Bias

A significant overall heterogeneity was noted in the study on hyperuricemia (*P* < 0.001, *I* ^2^ = 98%); however, the heterogeneity decreased in the subgroup analysis. We observed publication bias in both studies according to Egger's test. Then we performed a trim and fill method to address the problem of publication bias. However, it became unchanged after we applied the trim and fill method [65].

## 4. Discussion

We analyzed 44 epidemiological surveys covering 16 provinces, municipalities, and autonomous regions in mainland China. An important strength of our study is that it is a cross-sectional study. We systematically analyzed the prevalence of hyperuricemia and gout in mainland China. To our knowledge, this is the first study of this kind to focus on mainland China and cover the years from 2000 to 2014.

In our meta-analysis, the prevalence of hyperuricemia in mainland China was 13.3% (19.4% in men and 7.9% in women), which was in accordance with the worldwide prevalence rate reported to be ranging from 2.6% to 36% in different populations [66]. Our result was lower than that observed in several developed countries, such as the United States (21.2% in men and 21.6% in women) [21] and Japan (25.8% overall, 34.5% in men and 11.6% in women) [67]. As expected, the prevalence is close to that in most developing countries; for example, it is 10.6% in Thailand (18.4% in men and 7.8% in women) [68] and 12.1% in Turkey (19.0% in men and 5.8% in women) [69]. Chuang et al. performed the Nutrition and Health Survey in Taiwan (NAHSIT) study from 2005 to 2008, which focused on a Chinese population, but the results of their study differed significantly from those of our study. In their reports the prevalence of hyperuricemia was 21.6% in men and 9.6% in women [70], which was higher than ours and similar to that in developed countries. Our research was performed on mainland China, whereas Chuang's study was conducted in Taiwan, an economically-developed region in China. We believe that our results are more representative of the Chinese population living in the mainland.

As China is geographically vast, the prevalence of hyperuricemia varies significantly in different geographic regions. The prevalence in south China was 18.6%, which is much higher than the pooled prevalence, followed by southwest China (13.9%), north China (13.2%), east China (12.9%), northwest China (10.3%), and northeast China (10.1%). Such differences might be related to variability in lifestyle and economic development. As a previous study described, rapidly increasing economic development has led to unhealthy lifestyles [71]. Residents in south China, which is an economically developed region, consume more meat, seafood, and alcohol than residents elsewhere; therefore, the prevalence of hyperuricemia was higher in south China than in other regions. Also, hyperuricemia was more common in urban residents than in rural residents, and the inland prevalence of hyperuricemia was much higher than in coastal areas.

From our study, the pooled prevalence of gout was 1.1%, which is similar to that in Italy (0.9% in 2009) [19], France (0.9% in 2013) [72], the United Kingdom, and Germany (1.4% in 2000–2005) [17]. In addition, the prevalence of gout in our country was much higher than that in Turkey (0.31% in 2001-2002) [73], Mexico (0.3% in 2011) [74], Greece (0.47% in 2003) [75], and the Czech Republic (0.3% in 2002-2003) [76] but is markedly lower than that in New Zealand (2.69% in 2008-2009) [77], the USA (3.9% in 2007-2008) [21], and Australia (9.7% in 2002) [78].

Another main finding in our study was that the prevalence of gout in men (1.5%) was remarkably higher than in women (0.9%). This difference in sex was consistent with previous studies in other populations. Soriano et al. investigated the current epidemiology of gout in the general United Kingdom population and suggested that the incidence of gout was 4.42 per 1,000 persons per year in men and 1.32 per 1,000 persons per year in women [13]. Zhu et al. reported that the prevalence in the US was 5.9% in men, which was much higher than the 2.0% observed for women [21]. In accordance with these researches, prevalence of gout in Taiwan was 9.2% for men and 2.3% for women [70]. Sex hormones may explain the difference between the sexes. Ghei et al. suggested that the serum uric acid levels were higher in men than in women and that this difference is under the influence of sex hormones. Uric acid levels in women tend to increase after menopause [69, 79].

Moreover, in line with previous results, a rise in the prevalence of gout was observed in the current study. The prevalence was 0.9% in 2000–2005, 1.1% in 2006–2010, and 1.4% in 2011–2014. The US National Health and Nutrition Examination Survey (NHANES) study conducted in 2007-2008 demonstrated that the prevalence of gout was 3.9%, though it was only 2.7% in 1988–1994 [21]. The NAHSIT studies, carried out during 1993–1996 and 2005–2008, showed that the prevalence of gout increased from 4.7% to 8.2% in men and 2.2% to 2.3% in women [70]. To help reduce the increasing burden of these diseases, prospective data on modifiable risk factors in lifestyle and diet for these conditions should be considered including, but not limited to, weight control, regular exercise, restricted intake of meat and purine-rich foods, and avoidance of heavy drinking. Vitamin C supplementation may also be considered a long-term preventive measure as it can lower the risk of gout through lowering serum urate levels [80, 81].

Noteworthy, there is a lack of unified diagnostic criteria for gout, and several sets of criteria exist, such as the Rome criteria, the New York criteria, and the American Rheumatology Association (ARA) criteria [24]. The gold standard to diagnose gout is the presence of monosodium urate monohydrate (MSU) crystals in the synovial fluid (SF) at the time the patient experiences a gout attack [82]. The sets of criteria that include MSU crystals in SF have high specificity, and the exclusion of MSU crystal examination has led to a dramatic reduction in sensitivity [83]. However, MSU crystal examination is not always feasible in clinical practice. In 2015, Taylor et al. performed the Study for Updated Gout Classification Criteria (SUGAR) and determined ten parameters for accurately distinguishing gout from nongout [84]. In the same year, the American College of Rheumatology developed a new classification criteria for gout [85]. All the studies included in our analysis were performed from 2000 to 2014; therefore they were unable to adopt the new classification criteria. The diagnostic criteria used in this study could lead to a possible high sensitivity but low specificity. Because of this, the prevalence of gout in our analysis may be slightly higher than the actual rate, but it represents the general prevalence of gout and its geographical distribution in China.

Our meta-analysis has several other limitations. First, the pooled data covered only part of mainland China, especially for gout; however, our data did cover 16 provinces, municipalities, and autonomous regions. To our knowledge, it is the most encompassing cross-sectional study on hyperuricemia and gout prevalence in China. Second, the primary studies on hyperuricemia used different assays to assess serum uric acid levels with different reference intervals. Third, there were variations in the quality of the selected articles; hence heterogeneity may be influenced by uncertain data. Fourth, as much concern is given to this topic by Chinese doctors, the majority of the studies included were published in Chinese. However, this limitation was overcome by the current authors who are proficient in Chinese for interpretation and extraction of data. Also, sample size of included papers was too small in our subgroup analysis for the prevalence of gout, so there was no statistical power to explore the association between gout prevalence and geographic regions. Our work underlines the need for additional population-based investigations in the areas absent from our analysis. This is the first study to assess the nationwide epidemiology of hyperuricemia and gout in mainland China.

In conclusion, as previous studies were limited to specific regions, our study on the epidemiology of hyperuricemia and gout is of value to public health policies. Based on previous studies, we show that the prevalence of these diseases is high and that the rate of gout is rising. Consequently, large well-designed multicenter investigations are required in the future to provide information regarding the outcomes and prognosis of these chronic diseases in the entire population. Furthermore, effective measures should be adopted to prevent the increase in incidence of these diseases.

## Figures and Tables

**Table 1:** Characteristics of studies on the prevalence of hyperuricemia and gout.

First author	Publication year	Area	Diagnostic criterion (*μ*mol/L) (Men/Women)	Rural/urban	Inland/coastal	Study year	Sample size	Case	Prevalence (%)
Prevalence of hyperuricemia									
Shi [29]	2013	Shijingshan, Beijing	≥420/≥350	Urban	Inland	2012	3961	438	11.06
Ma [30]	2014	Xichengqu, Beijing	≥417/≥357	Urban	Inland	2012	834	100	11.99
Li [31]	2013	Bortala, Xinjiang	>420/>350	Rural	Inland	2009	2046	261	12.76
Zheng [32]	2010	Wenzhou, Zhejiang	≥417/≥357	Urban	Inland	2008	1520	114	7.50
Sun [33]	2008	Dalian, Liaoning	≥420/≥350	Rural	Coastal	2007	1024	100	9.77
Hou [34]	2010	Dalian, Liaoning	>420/>350	Rural	Coastal	2007	1021	97	9.50
Wang [35]	2010	Baoshan, Yunnan	>420/>350	Urban	Coastal	2009	1501	210	13.99
Yu [36]	2010	Foshan, Guangdong	≥417/≥357	Urban	Coastal	2008	7403	1117	15.09
Wu [37]	2008	Guangzhou, Guangdong	≥417/≥357	Urban	Inland	2007	2788	578	20.73
Zou [38]	2011	Guilin, Guangxi	≥420/≥360	Urban	Inland	2009	6273	1477	23.55
Wang [39]	2008	Zhoushan, Zhejiang	>420/>360	Rural	Inland	2007	1438	158	10.99
Meng [40]	2012	Gaoyou, Jiangsu	≥420/≥360	Rural	Inland	2010	4504	538	11.94
Shen [41]	2014	Wuxi, Jiangsu	≥417/≥357	Urban	Inland	2009	3723	754	20.25
Song [42]	2014	Nanchang, Jiangxi	>420/>350	Urban	Inland	2011	3795	795	20.95
Shao [43]	2003	Nanjing, Jiangsu	≥417/≥357	Urban	Inland	2003	7778	1038	13.35
Zhou [44]	2013	Ningbo, Zhejiang	>420/>370	Urban	Coastal	2008	2110	190	9.00
Huang [45]	2013	Ningbo, Zhejiang	>420/>360	Urban	Coastal	2012	1754	195	11.12
Xin [46]	2013	Qingdao, Shandong	>420/>350	Urban	Coastal	2011	5165	748	14.48
Tian [47]	2008	Qingdao, Shandong	>420/>350	Urban	Coastal	2006	2363	471	19.93
Tian [47]	2008	Qingdao, Shandong	>420/>350	Rural	Coastal	2006	2467	405	16.42
Dong [48]	2004	Qingdao, Shandong	>420/>350	Urban	Coastal	2002	2190	402	18.36
Zhang [49]	2006	Haiyang, Shandong	>416.36/>356.88	Rural	Coastal	2004	5372	649	12.08
Wang [50]	2010	Shenyang, Liaoning	>420/>350	Urban	Inland	2009	675	78	11.56
Chen [51]	2008	Chengdu, Sichuan	≥428	Urban	Inland	2006	2566	400	15.59
Guo [52]	2012	Taiyuan, Shanxi	≥420	Urban	Inland	2010	4228	371	8.77
Wang [53]	2010	Wenzhou, Zhejiang	>420/>350	Urban	Coastal	2008	3478	260	7.48
Shao [54]	2011	Wenzhou, Zhejiang	>420/>350	Urban	Coastal	2008	3480	260	7.47
Pan [55]	2014	Changzhou, Jiangsu	>420/>380	Rural	Inland	2008	3122	573	18.35
Duan [56]	2013	Korla, Xinjiang	>417/>357	Urban	Inland	2009	2046	261	12.76
Zhang [57]	2014	Xingtai, Hebei	>420/>350	Rural	Inland	2013	2109	177	8.39
Mou [58]	2013	Yantai, Shandong	≥380	Urban	Coastal	2012	635	66	10.39
Li [59]	2010	Yan'an, Shaanxi	>417/>357	Urban	Inland	2008	1290	71	5.50
Chen [60]	2009	Dali, Yunnan	>420/>350	Urban	Inland	2006	7505	923	12.30
Jin [61]	2009	Zhuhai, Guangdong	>420/>360	Rural	Coastal	2007	1112	164	14.75
Cai [62]	2009	Hangzhou, Zhejiang	>420/>360	Urban	Inland	2008	4155	702	16.90
You [63]	2014	Mongolian	≥416/≥357	Urban	Inland	2009	630	120	19.05
You [63]	2014	Mongolian	≥416/≥357	Rural	Coastal	2009	179	23	12.85
Zhang [64]	2011	Tianjin	>420/>360	Urban	Coastal	2009	17762	2160	12.16

Prevalence of gout									
Yu [36]	2010	Foshan, Guangdong	—	Urban	Coastal	2008	7403	77	1.04
Wu [37]	2008	Guangzhou, Guangdong	—	Urban	Inland	2007	2788	40	1.43
Song [42]	2014	Nanchang, Jiangxi	—	Urban	Inland	2011	3795	58	1.53
Shao [43]	2003	Nanjing, Jiangsu	—	Urban	Inland	2003	7778	105	1.35
Zhang [49]	2006	Haiyang, Shandong	—	Rural	Coastal	2004	5372	23	0.43
Zhang [57]	2014	Xingtai, Hebei	—	Rural	Inland	2013	2109	26	1.23

**Table 2:** Gout classification criteria.

Yu et al. [36]	Wu et al., Song et al., Shao et al., Zhang et al., and Zhang et al. [37, 42, 43, 49, 57]
Classification criteria for gout [25] (1) More than one attack of acute arthritis (2) Maximum inflammation developed within 1 day (3) Oligoarthritis attack (4) Redness observed over joints (5) First MTP joint painful or swollen (6) Unilateral first MTP joint attack (7) Unilateral tarsal joint attack (8) Tophus (suspected or proven) (9) Hyperuricemia (more than 2 S.D. greater than the normal population average) (10) Asymmetric swelling within a joint on X-ray (11) Complete termination of an attack Case definition: ≥6 of 11 clinical criteria	ARA preliminary classification criteria for acute gout 1977 [24] (1) More than one attack of acute arthritis (2) Maximum inflammation developed within 1 day (3) Oligoarthritis attack (4) Redness observed over joints (5) First MTP joint painful or swollen (6) Unilateral first MTP joint attack (7) Unilateral tarsal joint attack (8) Tophus (suspected or proven) (9) Hyperuricemia (more than 2 S.D. greater than the normal population average) (10) Asymmetric swelling within a joint on X-ray (11) Subcortical cysts without erosions on X-ray (12) Complete termination of an attack Case definition: ≥6 of 12 clinical criteria required or presence of MSU crystals in SF or in tophus.

**Table 3:** Stratified prevalence of hyperuricemia in mainland China.

Subgroups	Prevalence (%) (95% CI)	Number of studies	Heterogeneity		Case/total
			*I* ^2^%	*P* value	
Area					
Urban	13.7 (12.0, 15.4)	27	98.4	<0.001	14322/101787
Rural	12.3 (10.5, 14.1)	11	94.3	<0.001	3154/24581
Coastal/inland					
Inland	13.8 (11.8, 15.7)	23	98.3	<0.001	10160/68666
Coast	12.5 (10.8, 14.2)	15	97.3	<0.001	7316/57702
Location					
North China	13.2 (11.5, 14.8)	13	96.3	<0.001	6162/48261
East China	12.9 (10.2, 15.6)	12	98.6	<0.001	5577/40857
Northwest	10.3 (5.4, 15.3)	3	97.4	<0.001	593/5382
Northeast	10.1 (8.9, 11.2)	3	0.0	0.376	275/2720
Southwest	13.9 (11.7, 16.1)	3	88.6	<0.001	1533/11572
South China	18.6 (13.8, 23.3)	4	98.3	<0.006	3336/17576
Economic level					
High	13.8 (12.0, 15.6)	20	98.0	<0.001	8094/59811
Low	12.6 (10.6, 14.7)	18	98.1	<0.001	9382/66557
Sex					
Male	19.4 (17.6, 21.1)	38	96.7	<0.001	11644/60768
Female	7.9 (6.6, 9.3)	38	97.9	<0.001	5859/65654
Total	13.3 (11.9, 14.6)	38	98.0	<0.001	17476/126368

**Table 4:** Prevalence of gout in mainland China by different stratification factors.

Subgroups	Prevalence (%) (95% CI)	Number of studies	Heterogeneity		Case/total
			*I* ^2^%	*P* value	
Area					
Urban	1.2 (0.7, 1.8)	4	0.0	0.830	280/21764
Rural	0.9 (0.2, 1.6)	2	14.0	0.313	49/7481
Coastal/inland					
Inland	1.4 (0.8, 1.9)	4	0.0	0.989	229/16470
Coastal	0.8 (0.2, 1.4)	2	0.4	0.316	100/12775
Study year					
2000–2005	0.9 (0.0, 1.8)	2	59.1	0.118	128/13150
2006–2010	1.1 (0.4, 1.8)	2	0.0	0.655	117/10191
2011–2014	1.4 (0.5, 2.2)	2	0.0	0.737	84/5904
Sex					
Male	1.5 (0.8, 2.1)	6	1.9	0.404	226/14060
Female	0.9 (0.0, 1)	6	0.0	0.924	78/15185
Total	1.1 (0.7, 1.5)	6	0.0	0.644	329/29245
